# A unified framework of demographic time

**DOI:** 10.1186/s41118-017-0024-4

**Published:** 2017-08-22

**Authors:** Tim Riffe, Jonas Schöley, Francisco Villavicencio

**Affiliations:** Max-Planck-Institut für Demografische Forschung, Rostock, MV Germany

**Keywords:** Age structure, Formal demography, Data visualization, Age period cohort

## Abstract

Demographic thought and practice is largely conditioned by the Lexis diagram, a two-dimensional graphical representation of the identity between age, period, and birth cohort. This relationship does not account for remaining years of life, total length of life, or time of death, whose use in demographic research is both underrepresented and incompletely situated. We describe an identity between these six demographic time measures and describe the sub-identities and diagrams that pertain to this identity. We provide an application of this framework to the measurement of late-life morbidity prevalence. We generalize these relationships to higher order identities derived from an arbitrary number of events in calendar time. Our examples are based on classic human demography, but the concepts we present can reveal patterns and relationships in any event history data, and contribute to the study of human or non-human population dynamics measured on any scale of calendar time.

## Introduction

In the course of training, all demographers are introduced to the Lexis diagram, a convenient graphical identity between the three main time measures used to structure demographic stocks and flows: age, period, and birth cohort. This representation does not account for time of death, time until death, or length of life, which may be of interest to researchers as structuring rather than latent variables in order to capture variation in demographic data.

We wish to draw attention to three time indices that are complementary to age (A), period (P), and birth cohort (C). The first such index is time to death, which we refer to as “thanatological age” (T) in contrast to “chronological age” (A). The second index is death cohort (D), which groups all individuals (of different ages) dying in the same time period. Finally, lifespan (L) or individual age-at-death itself is an index by which data may be structured. We therefore have six time measures in total to relate. We call these *measures of demographic time* because each, except period, depends on the timing of birth, death, or both.

The Lexis diagram can be understood as an APC plane that relates age, period, and birth cohort. Other such planes are also identifiable. The “thanatological dual” of APC is an identity between thanatological age, period, and death cohort, TPD. A third identity relates thanatological age, chronological age, and lifespan, TAL. A fourth identity relates lifespan, birth cohort, and death cohort, LCD. Each of these four “triad identities” (APC, TPD, TAL, and LCD) is sufficiently described by any two of its constituent indices. For instance, if the exact age of an individual at a particular time is known, the birth cohort to which he or she belongs can be immediately derived. Each of these four identities also lacks a major dimension of time. The TAL identity lacks calendar time, the LCD identity is ageless, APC lacks an endpoint in time, and TPD lacks a starting point in time. To our knowledge, the only triad identity that has received serious treatment at the time of this writing is the APC identity. Different aspects of the APC identity have been discussed since at least 1868 (Knapp 1868), and discussion remains lively today. Here, we relate the six major indices of time in a geometric identity, in much the same spirit as the work on APC relationships done between the late 1860s and mid 1880s^1^.

Our goal is to describe the geometric identity between all six primary measures of demographic time, the identity *unifying* the four aforementioned triad identities: a hexad identity among A, P, C, T, D, and L. This novel identity may be useful or an intuitive referent for demographers in the same way as the Lexis diagram is. We also give a bottom-up description of how temporal identities all arise from the notion of distinct events situated in time and the durations separating them. These more general event-duration foundations facilitate comparison of our proposed demographic time framework with other temporal designs found in the literature, such as the disease duration space of Brinks et al. (2014), or the marriage identity described by Lexis (1875). The framework we describe is general and adaptable for any event history scenario, and it is useful as a system for delineating and deriving the full set of temporal implications in a given dataset. In this way, our system may serve as a reference for temporal statistical designs, useful both for relating different models and for expanding a given design to its full-time consequences.

Just as the Lexis diagram is a fundamental instrument to teach demography, we hope that the demographic time measures and their graphical depictions presented here will be helpful to teachers and young demographers who wish to explore time structures beyond age, period, and cohort. The temporal relationships we describe will also be useful for researchers to better detect and understand patterns in their data and for methodologists to rigorously account for the structure of data in demographic methods or statistical designs. Substantively, the concepts we present are applicable to the structure and study of any phenomenon or transition that varies in time, including single or multistate processes.

We begin by defining some terms used throughout the manuscript. We then explore all combinations of two time measures, the dyadic relationships, followed by the four triad identities and their diagrams, a generalization of the Lexis diagram to *n*-dimensional space, and finally, we present the hexad demographic time-identity.

## Definitions

### Technical terminology

The following list describes some of the more important terms we use. **Demographic time measures** are any of the six time indices discussed to describe demographic time: chronological age (A), period (P), birth cohort (C), thanatological age or time to death (T), lifespan or age-at-death (L), and death cohort (D). **Dyads, triads, and hexads** are any set of two, three, or six unique time measures, respectively. **A triad identity** is a triad with the property that each of its members can be derived from the other two with no additional information. There are four triad identities: APC, TPD, TAL, and LCD. **A temporal plane** is any (*x,y*)-mapping of a dyad of time measures.

Using this terminology, we say that the “Lexis” measures constitute a triad identity between chronological age, period, and birth cohort. Each dyad combination of elements in this identity can be mapped to a temporal plane, the Lexis diagram. If we know that Mindel turned 50 on the 21st of May, 1963, then we also can derive that she was born on the 21st of May, 1913. Hence, any two pieces of information in this case will give the third, and the same holds for the other triad identities.

### Time measures

We describe time in terms of years, the dominant time scale for human demography, although all relationships are scalable to any time unit. We therefore refer to calendar time. We also describe the framework in terms of human lifespans, although it applies in a more general sense to any durations observed over time. This is to say, birth may be translated to entry, and death to exit, or any other absorbing state. The six measures of time we consider are defined in Table 1, both in the demographic sense we describe, as well as in a more general event history interpretation.

**Table 1:** Definitions of the six time measures

Time measure	Demographic definition	Event history definition
A—chronological age	Time since birth	Time since start of exposure
P—period	Calendar time	Calendar time
C—birth cohort	Calendar time of birth	Calendar time of exposure start
T—thanatological age	Time until death	Time until event
D—death cohort	Calendar time of death	Calendar time of event
L—lifespan	Duration of life	Duration of exposure

The concepts of thanatological age and death cohorts are likely less familiar to readers than the other measures we consider. Thanatological age is remaining time until death, the information approximated with life expectancy. This term is sometimes referred to in the literature as life left, time to death, remaining lifespan, follow-up duration, residual life, or reverse time. Chronological and thanatological age are in this way complementary, duals, and birth, and death cohorts are a similar kind of dual. Cohorts in general associate individuals that share a characteristic, often a combination of place and time. The deaths of a given year are not usually referred to as a death cohort, although this concept was already introduced by (Brouard 1986) as “génération de décès” in a retrospective study of the French population from the twentieth century. In the time preceding death, the members of a given death cohort likely have much in common, despite heterogeneity with respect to time of birth. In event history or non-human contexts, anologs to death cohorts in this framework may be even more meaningful.

Much of the work of demography is directed at the study of lifespan. Lifespan is synonymous both with longevity, chronological age at death, and thanatological age at birth. One’s ultimate completed lifespan is constant throughout life, though we have no knowledge of it until death: It is assigned retrospectively. Demographers have more often used lifespan or age-at-death as a measure of mortality, or similar, than as a measure on which to compare individuals or structure data.

Treating lifespan, death cohorts, and thanatological age as temporal structuring variables enables new classes of comparisons, models of understanding, and discovery, akin to those unlocked by breaking down demographic phenomena by chronological age, period, and birth cohort. The following sections, in this sense, provide an exhaustive classification of the ways in which these six measures of time can be juxtaposed to such ends.

## From dyads to the triad identities

We distinguish between two kinds of dyads: informative dyads and uninformative dyads. Informative dyads are any pair of measures from which a third time measure can be derived, forming a triad identity. There are $15=\binom {6}{2}$ possible dyads in our set of time measures, 12 of which are informative, and 3 of which have no derived time measure, and are therefore called uninformative. For instance, if we take the dyad TA, L is the derived measure, and TAL the corresponding triad identity. In contrast, nothing can be derived from the LP dyad: One can have an eventual lifespan of 100 in the year 2016 and still be alive with the same eventual lifespan in 2017.

In this section, we systematically map each dyad to its temporal plane, and we synthesize these into the four primary identities and their essential diagrams. We render the 15 dyad-based diagrams that can be derived from the six time measures. Of these 15, 12 diagrams can be distilled into just four, the triad identity diagrams. Each triad identity diagram is then briefly discussed with suggested or speculated applications.

### The question of mapping

Any mapping of two time measures to an (*x,y*) coordinate system constitutes a temporal plane. If the two given time measures are members of the same triad identity, the third member is a derived measure. If we assign A to *y* and P to *x*, thereby implying C (and the APC triad identity), we state this relationship explicitly by writing AP(C). The temporal plane that corresponds to this informative dyad is the contemporary representation of the Lexis diagram (Lexis 1875; Pressat 1961). The informative dyads AC(P) and CP(A) also belong to the Lexis identity but imply different less-common rotations and projections of the Lexis diagram.

For each dyad, there is a fundamental question of how to map the constituent coordinates to a Cartesian temporal plane. Typically, one forces parity between time units within a specified dyad, mapping one element directly to *x* and the second element directly to *y*, resulting in a 90° angle between the *x* and *y* axes. In this case, it is conventional to force a unity aspect ratio between the *x* and *y* axes, such that the derived measure, if any, is then *accidentally* present in a 45° ascending or descending angle, depending on the dyad and axis orientation.

It has long been noted (Lexis 1875; Perozzo 1880) that the derived time measure (usually birth cohort) is longer than either the age or period axes when plotted at 45°. If a right angle and unity aspect ratio is forced between the dyad, the derived measure is always stretched by $\sqrt {2}$. Another logical mapping would be to translate to (*x,y*) coordinates that force 60° angles between the three measures. Such a mapping ensures that the spatial units are equal for the three measures, and we therefore refer to it as the isotropic mapping. The isotropic mapping is comparable to using ternary or barycentric coordinate systems: The three variants of each triad identity are simple rotations of one another, and they require no rescaling. The primary justification for isotropic demographic surfaces comes from a data visualization perspective, where it may be hypothesized that the viewer’s ability to compare slopes is hindered if time coordinates are not on the same scale. For the sake of clarity, all two-dimensional diagrams are rendered in Cartesian rather than isotropic coordinates.

### Dyads to diagrams

Each of the 15 dyads, an explanation or simple example, and the corresponding diagram representations are summarized in Table 2. The 12 informative dyads consist of two elements from one of the four triad identities (APC, TPD, TAL, LCD), which we analyze in detail in further sections. The uninformative dyads are simply pairs of time measures that do not have a derived measure and therefore are not contained in any of these four triad identities.

**Table 2:** All dyadic juxtapositions of the six measures of demographic time

Variants of APC		
AP(C) C = P − A	The AP(C) temporal plane constitutes the classical Lexis diagram.	
AC(P) P = C + A	The AC(P) temporal plane is equivalent to the Lexis diagram except birth cohort is given and period is derived rather than the other way around.	
CP(A) A = P − C	The CP(A) temporal plane is equivalent to the Lexis diagram except birth cohorts are given and age is derived rather than the other way around.	
Variants of TPD		
TP(D) D = P + T	Helen had 30 years of life left (T) in 1971 (P) and therefore belonged to the 2001 death cohort (D)	
PD(T) T = D − P	Mindel died in 1973 (D). In 1953 (P) she had 20 years left to live (T).	
TD(P) P = D − T	Irene died in 1974 (D). When she had 30 remaining years of life (T) the year must have been 1944 (P).	
Variants of TAL		
TA(L) L = T + A	The time already lived and the time still left sum up to the total lifespan.	
TL(A) A = L − T	Helen lived to the age of 86 (L). When she had 20 years left (T) she must have been 66 (A).	
AL(T) T = A − L	Tim is 34 years old (A) and will live to the age of 96 (L), leaving him 62 years (T) to settle affairs.	
Variants of LCD		
LC(D) D = C + L	Àngels was born in 1940 (C) and she lived to be 64 (L), implying an untimely death in 2004 (D)	
CD(L) L = D − C	Pascal was born in 1893 (C) and died in 1964 (D), implying a lifespan of 71 (L), or so.	
LD(C) C = D − L	Margaret died in Dec., 1995 (D) with a completed lifespan of 96 (L), putting her birth year in 1900 (C).	
The uninformative dyads		
LP(-)	The LP plane is *non-informative*. No additional measures can be derived knowing just lifespan and period.	
CT(-)	The CT plane is *non-informative*. No additional measures can be derived knowing just birth cohort and thanatological age.	
AD(-)	The AD plane is *non-informative*. No additional measures can be derived knowing just death cohort and age.	

Most of what we know about how rates change over age and time comes from the very first juxtaposition in Table 2, AP(C). While CP(A) and AC(P) are statistically redundant when exact times are used, they are not fully redundant if based on discrete double-classification of data, as often provided in aggregated official statistics. Double classified data are found on the APC diagram in the shape of squares (AP), horizontal parallelograms (AC) and vertical parallelograms (CP), and these are commonly used to compute different kinds of demographic rates and probabilities (Caselli et al. 2006, p. 63). The other dyadic juxtapositions (involving the measures T, D, or L) can be considered as either rare or novel ways of structuring or viewing temporal variation in demography, and these imply new families of rates and probabilities.

### The triad identities

There are $20=\binom {6}{3}$ ways to choose three different time indices out of six, of which four form a triad identity: APC, TPD, TAL, and LCD. Given the three time measures from any of the triad identities, one can derive no further time measures. If one selects three random time indices that do not form any of these four triad identities (20−4=16 possibilities), this property does not hold. For instance, in the triad APT, age and period are not sufficient to determine thanatological age. Given the triad APT, one can however derive the remaining three time measures.

Triad identities are more meaningful than uninformative dyads. This is so even in the absence of data, due to the underlying relationship between measures. Each of the triad identities can accommodate some version of a lifeline, for instance. In the following, we therefore lay out the four primary diagrams that belong to the triad identities. The question of which diagram mapping is relevant to a given demographic phenomena is a function of patterns in the data. The best diagram is the one that captures all meaningful variation in the data. If APC highlights meaningful variation in a phenomenon, then its representation as such is useful, and the same holds for the other identities.

#### APC: chronological age, period, and birth cohort

The Lexis diagram has long been used in demography as a conceptual tool for structuring data, observations, and rate estimation, as inspiration for work on statistical identification, and as the coordinate basis of contemporary Lexis-surfaces. Since the Lexis diagram could have been named for others (Keiding 2011; Vandeschrick 2001), and since we compare with other temporal configurations, we refer to it as the APC diagram.

**Fig. 1:**
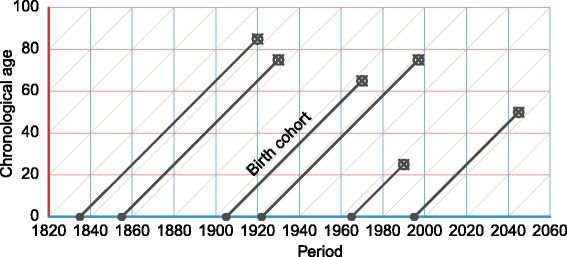
An APC diagram with six lifelines

The APC diagram in Fig. 1 represents years lived on the *y*-axis, calendar years on the *x*-axis, and birth cohorts as the right-ascending diagonals. This is the most common of several possible configurations of the APC dimensions. Individual lifelines (black) are aligned in the birth cohort direction, starting with birth (filled circle) at chronological age zero, and death (circled x). Any APC surface can be interpreted along each of these three dimensions of temporal structure.

#### TPD: thanatological age, period, and death cohort

**Fig. 2:**
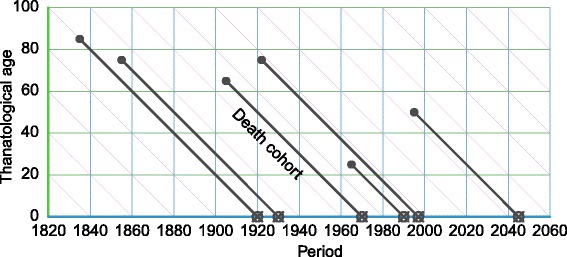
A TPD diagram with six lifelines

The TPD diagram is best imagined as the inverse of the APC diagram. One may take the same individuals represented in Fig. 1 and group them by death cohorts (D) instead of birth cohorts (C). Lifelines then descend such that all endpoints align to thanatological age 0, creating the diagram in Fig. 2 in which individuals dying at different ages but in the same time period are grouped together. To our knowledge, the TPD diagram has only appeared once in the literature, as a didactic aid in a proof of symmetry between chronological and thanatological age structure in discrete stationary populations (Villavicencio and Riffe 2016). TPD diagrams may also be useful to arrange events or durations that are logically aligned (or may only be aligned) by time of termination. It may be reasonable to align on termination in cases where this brings preceding patterns of variation into focus.

There are several examples of analysis of this kind of data, usually stemming from a lack of information on chronological age. This is the case, for instance, in biodemographic studies in which wild animals with unknown ages are captured and then followed up until death (Müller et al. 2004, 2007). Other examples are human historical databases, which usually lack information about births, but individuals can be traced from a particular event until death. This is the case in the Barcelona Historical Marriage Database, which collects information about marriage licenses of Barcelona (Spain) from the mid-fifteenth century until the early twentieth century. In this database, ages are unknown, but individuals are first identified in their marriage record and an estimation of the times of death is plausible (Villavicencio et al. 2015). We speculate that TPD diagrams could also be used in biomedical studies for the representation of lifelines preceding deaths from infectious or acquired conditions, when the time of infection or acquisition remains unknown, an issue which has received attention in the statistical literature (Chan and Wang 2010).

#### TAL: thanatological age, chronological age, and lifespan

TAL is an appropriate diagram to examine processes that vary over the life course. More precisely, the TAL plane can highlight variation that is related to time since birth, time until death, length of life, and their combinations. These key aspects of demographic time are compressed to chronological age only in the APC perspective, which can blend out meaningful variation. Since the life course belongs to the cohort perspective, it is best to think of the TAL plane as belonging to some particular birth cohort. Alternatively, a TAL triangle may be taken as a cross-section through the period dimension, a sort of synthetic TAL plane.

**Fig. 3:**
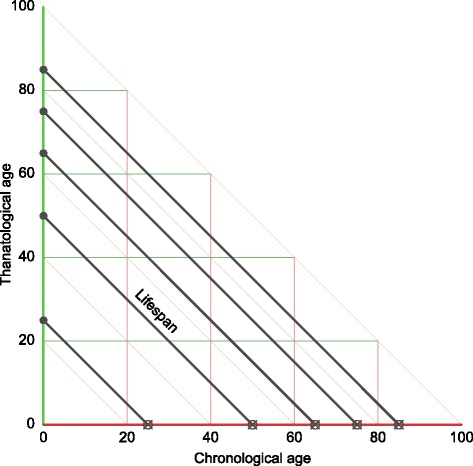
A TAL diagram with six lifelines. Since two of the six lifelines are of equal length (75), they are overlapped in this figure and appear to be five

To our knowledge, the TAL diagram has only appeared once in the literature, in an exploration and classification of late-life health conditions (Riffe et al. 2016). There are however instances of statistical designs adapted to this coordinate plane (McCullagh and Dempsey 2013; Jewell 2016). The TAL diagram in Fig. 3 contains no indication of period or cohorts, as calendar time is blended out in this diagram. The lifelines depicted are identical to those shown in APC Fig. 1 and TPD Fig. 2. The TAL diagram is useful for characterizing patterns of prevalence of health conditions. We speculate that data structured and aligned in this way may yield hitherto undescribed patterns in other contexts, e.g., measurements on mother and fetus over the course of a pregnancy may vary by age of gestation, time until parturition, or total length of the pregnancy; growth and reproduction patterns over age may be conditioned on the life-span of an organism.

#### LCD: lifespan, birth cohort, and death cohort

**Fig. 4:**
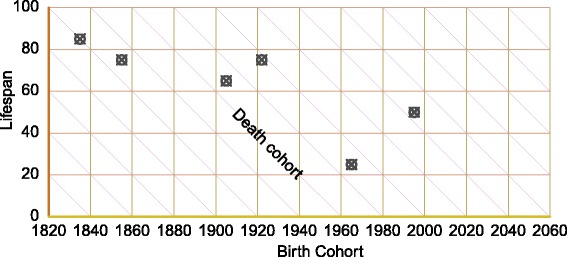
An LCD diagram with six lifelines. Since the LCD plane is orthogonal to the life course, lifelines are depicted as points

The LCD diagram completes our set of identities. It is based on the relationship between lifespan, birth cohort, and death cohort. In Fig. 4, lifespans are indexed by the *y*-axis, while birth cohorts are indexed by the *x*-axis, and death cohorts are found in descending diagonals. To structure data on these three time measures implies excluding time-varying information over the life course. An individual only ever has one lifespan, one birth cohort, and one death cohort, such that the LCD coordinates of an individual are constant throughout life. The LCD plane is therefore orthogonal to lifelines, and individuals are located with points, rather than life segments. In Fig. 4, the same six individuals from previous diagram figures are represented with crossed circles.

We recommend this mapping for plotting surfaces of values that are cumulative or static over the life course, but that may vary over time or by length of life. Imagine an LCD surface of cumulative life course consumptive surplus or deficit, or anything else that might vary by lifespan and moment of birth or death, such as children ever born, years of retirement, the size of trees or other aspects of forestry, populations of buildings in large cities, and so forth. Lexis (1875) describes an analogous relationship between marriage cohort, separation cohort, and duration of marriage.

## The relationship between events and durations

The four identity-based diagrams discussed in prior sections are likely straightforward, either because the Lexis diagram is already familiar to the reader, or because Cartesian representations are widely used. However, the special relationship between these diagrams is based on a single hexad identity, which is less straightforward, and its resultant diagram is best derived from a more general groundwork. In this section we therefore describe a more general approach to understanding and constructing higher order temporal identities. This approach is based on a categorization of time measures into events and durations, and the realization that durations derive from events in calendar time.

### A general framework

The general relationship between events and durations serves not only to introduce the full demographic time framework but also to compare it with other relatively complicated temporal designs in the literature. Each of the six time measures that we have treated can be categorized into two basic types: events and durations. Events include birth (C) and death (D) cohort, as well as period itself (P). Durations are time differences between pairs of events: chronological age A = P – C, thanatological age T = D – P, and lifespan L = D – C. In the following we describe APC, APCTDL and other time frameworks in terms of vector spaces which, via linear transformation, relate the timing of events with durations between events.

#### **Definition 1**

Let ***p***=(*p*_1_,…,*p*_*n*_)^⊤^∈**R** ^*n*^ be a vector of *n* events or points in time with *n*≥2. A corresponding vector of durations ***d***∈**R** ^*m*^ is composed by elements of the form *d*_*i,j*_=*p*_*j*_−*p*_*i*_ for *i*=1,…,*n*−1, *j*=2,…,*n* and *j*>*i*.

The vector of events ***p*** can be ordered in an arbitrary way as long as the same elements in ***p*** correspond to the same type of event for all observations. A consequence of this is that durations may be either negative or positive depending on the ordering of events over the life course.

#### **Proposition 1**

Given a vector of events ***p***=(*p*_1_,…,*p*_*n*_)^⊤^∈**R** ^*n*^, the dimension of the corresponding vector of durations ***d***∈**R** ^*m*^ is *m*=*n*(*n*−1)/2.

#### *Proof*

By definition, each element of ***d*** is formed by two different elements of ***p***. Therefore, the length of ***d*** is the number of combinations of 2 different elements from a set of size *n*, such that the order of selection does not matter. From combinatorial theory, it is well known that this value is given by the binomial coefficient $\binom {n}{2}=\frac {n!}{2!(n-2)!}=n(n-1)/2$. □

#### **Proposition 2**

For any vector of events ***p***=(*p*_1_,…,*p*_*n*_)^⊤^∈**R** ^*n*^, there is always a linear transformation *f*:**R** ^*n*^→**R** ^*m*^ that provides a corresponding vector of durations ***d***∈**R** ^*m*^.

#### *Proof*

The existence of *f* is a direct consequence of Definition 1, given that all the elements of ***d*** are a linear combination of elements of ***p***. □

#### **Corollary 1**

Given ***p***=(*p*_1_,…,*p*_*n*_)^⊤^∈**R** ^*n*^, suppose that ***d***=(*p*_2_−*p*_1_,…,*p*_*n*_−*p*_1_,*p*_3_−*p*_2_,…,*p*_*n*_−*p*_2_,…,*p*_*n*_−*p*_*n*−1_). Then, the linear transformation *f*:**R** ^*n*^→**R** ^*m*^ that yields ***d*** from ***p*** is defined by the *m*×*n* matrix



such that ***d***=***X***×***p***, and where *I*_*k*_ denotes the *k*×*k* identity matrix.

These results imply that given an arbitrary set of *n*≥2 points in time, it is always possible to calculate the durations between any pair of these points. However, note that matrix ***X*** in (1) yields a vector of durations ***d***∈**R** ^*m*^ whose elements are sorted in an arbitrary way. The following statement may be relevant in this regard.

#### **Proposition 3**

Given a vector of events ***p***=(*p*_1_,…,*p*_*n*_)^⊤^∈**R** ^*n*^, the corresponding vector of durations ***d***∈**R** ^*m*^ is unique, irrespective of the sorting of its elements.

#### *Proof*

Let us suppose that ***d*** ^***1***^ and ***d*** ^***2***^ are two different vectors of durations corresponding to the same vector of events ***p***∈**R** ^*n*^. Provided that ***d*** ^***1***^ and ***d*** ^***2***^ are finite and, by definition, both have dimension *m* and are formed by the same combinations of elements of ***p***, it will always be possible to re-arrange the elements of ***d*** ^***2***^ in the same order as ***d*** ^***1***^ such that ***d*** ^***1***^=***d*** ^***2***^. □

This last proposition allows considering ***X*** as the matrix defining the linear transformation between points and durations. Given a vector ***p*** and the corresponding ***d***=***X***×***p***, any differently sorted vector of durations would be obtained by swapping the rows of ***X***. Further, note that ***X*** does not have an inverse matrix, and therefore there is no linear transformation from durations to events. This is intuitively straightforward if one thinks that two vectors of events can yield the same vector of durations. In other words, a particular vector of durations can come from infinite different vectors of points in time. For instance, using ***X***, the vectors of events ***p*** ^***1***^=(1,2,3) and ***p*** ^***2***^=(2,3,4) both yield ***d***=(1,2,1). With respect to the six time measures discussed here, note that the events CPD yield TAL, but TAL does not yield CPD.

The relationship between events and durations can be systematically represented in a series of timelines and graphs that may better guide intuition. The joint relationship between events and durations is more explicit and more compact in a graph representation. As introduced in the following definition, the total number of time measures implied by a set of *n* events and the corresponding durations is *n*+*m*=*n*+*n*(*n*−1)/2=*n*(*n*+1)/2.

#### **Definition 2**

Given a vector of events ***p***=(*p*_1_,…,*p*_*n*_)^⊤^∈**R** ^*n*^, *n*≥2, and the corresponding vector of durations ***d***∈**R** ^*m*^, we define the graph of time measures ***G*** as the graph with *n*+*m*=*n*+*n*(*n*+1)/2 edges labelled by (***p***,***d***)∈**R** ^*n*(*n*+1)/2^ such that the relationships in Definition 1 are preserved.

Table 3 displays a timeline and a graph for two, three, and four event sets. The central column shows timelines, a familiar linear representation of time, with events marked with red ticks labelled with *p*_1_…*p*_*n*_. Durations span each of the *m* possible event dyads and are drawn below the main timeline as curly braces labelled with *d*_1,2_…*d*_*n*−1,*n*_. The right column of Table 3 draws the corresponding graph with a total of *n*+1 vertices and *n*+*m*=*n*(*n*+1)/2 edges for the elements of both ***p*** and ***d***. All events of ***p*** connect to a single vertex, and event edges are indicated in red with red-circled labels. In this rendering, each triangle formed by three mutually connecting edges represents a triad identity. The top row *n*=2 consists in a single identity. Three and four events imply a total of four and ten triad identities, respectively, and in general a given higher order identity will yield $\binom {n+1}{3}$ triad identities. We call this a temporal plane graph because the triangle resulting from any given triad sub-identity can be extended over all valid values of its time measures to form a temporal plane, as of the diagrams in “The triad identities” section. The dimensionality of the extended diagram of a given identity follows from the number of events from which the identity is derived: *n*=2 produces a two-dimensional diagram, *n*=3 produces a 3-dimensional diagram, and so forth.

**Table 3:** Event-duration timeline and graph for two, three, and four event sequences

#### **Definition 3**

We define *P*⊆**R** ^*n*^ as the vector-space (event-space) spanning all possible values vector ***p*** may take, and *D*⊆**R** ^*m*^ as the vector space spanning all possible instances of the duration vector ***d***.

Just like the APC diagram allows for *all possible* combinations of period, cohort and age we may consider the vector space *P* spanning all possible instances of ***p***. The calculation of durations between events as described in Proposition 2 can then be understood as a linear transformation from a vector space *P* whose bases represent events to a duration vector space *D* whose bases represent durations.

### Examples

The following examples show how different demographic time frameworks can all be expressed as instances of the event-duration vector space defined above.

**Example 1: The Lexis surface**

Let ***p*** have two elements, as in the first row of Table 3. Then ***d*** consists of just one element, defined as

1 $$ d_{1,2} = p_{2} - p_{1} \quad\quad.  $$

Interpreting *d*_1,2_ as *age*, *p*_2_ as *period*, and *p*_1_ as *birth cohort* yields the APC identity. The standard Lexis surface is constructed via a change of basis from the event-space *P*, featuring basis vectors (*p*_1_,*p*_2_), to the event-duration space *M*, featuring basis vectors (*p*_2_,*d*_1,2_).

**Example 2: Lexis’ marriage identity**

Along with his well known 2-dimensional diagram Lexis (1875) also described a 3-dimensional extension applied to the marriage and separation processes, reproduced in Keiding (2006). Let ***p*** have three elements, as in the second row of Table 3. Then ***d*** is defined as

2 $$ \begin{aligned} d_{1,2} = p_{2} - p_{1}\\ d_{1,3} = p_{3} - p_{1}\\ d_{2,3} = p_{3} - p_{2} \end{aligned} \quad\quad.  $$

Interpreting *p*_1_ as *birth cohort*, *p*_2_ as *marriage cohort* and *p*_3_ as *separation cohort* yields the durations *d*_1,2_ as *age at marriage*, *d*_1,3_ as *age at separation*, and *d*_2,3_ as *duration of marriage*. Lexis’ “marriage space” *M* is reconstructed by a change of basis from *P*⊆**R** ^3^→*M*⊆**R** ^3^, with the new orthogonal basis formed by (*p*_1_,*d*_1,2_,*d*_2,3_).

**Example 3: Adding death cohort to the Lexis surface**

As in Example 2 we start with a three element vector ***p*** yielding the very same identities as in Eq. (2) and the second row of Table 3, but with different interpretations. Interpreting *p*_1_ as *birth cohort*, *p*_2_ as *period* and *p*_3_ as *death cohort* yields the durations *d*_1,2_ as *chronological age*, *d*_1,3_ as *lifespan*, and *d*_2,3_ as *time to death*. This vector space contains the Lexis surface as a sub-space, as well as the other planes presented in “The triad identities” section. We return to this identity in the following sections.

**Example 4: Brinks’ Illness-Death model**

Brinks et al. (2014) describe an illness-death process atop the Lexis surface, and with diagnosis and death as additional events, for a total of four events. Let ***p*** have four elements, as in the third row of Table 3. Then ***d*** is defined as:

3 $$ \begin{aligned} d_{1,2} = p_{2} - p_{1}\\ d_{1,3} = p_{3} - p_{1}\\ d_{2,3} = p_{3} - p_{2}\\ d_{1,4} = p_{4} - p_{1}\\ d_{2,4} = p_{4} - p_{2}\\ d_{3,4} = p_{4} - p_{3} \end{aligned} \quad\quad.  $$

Interpreting *p*_1_ as *birth cohort*, *p*_2_ as *period*, *p*_3_ as *time at diagnosis*, and *p*_4_ as *death cohort* yields the following composition of ***d***: *d*_1,2_ is *chronological age*, *d*_1,3_ is *age at diagnosis*, *d*_1,4_ is *lifespan*, *d*_2,3_ is *time to/since diagnosis* ^2^, *d*_2,4_ is *time to death*, and *d*_3,4_ is duration of illness (an irreversible state).

Unlike the other examples, the actual vector-space of demographic time shown in Brinks et al. (2014) Fig. 2 only identifies a *subset* of the times-measures implied by the model of the authors, namely age (*y*-axis), period (*x*-axis), birth cohort (implied by a linear combination of age and period) and duration of disease (*z* axis). Although lifelines in this depiction only begin to ascend into the disease duration axis at the time of disease diagnosis, this event time measure is not ascribed to an axis per se or implied by the other axes, and no further time scales can be derived from the three axes drawn. Instead, a few additional events and durations (death cohort, timing of diagnosis and duration of disease) are introduced as *markings* within the three dimensional vector space, just as one would mark *specific* life-lines on a Lexis-diagram without accounting for *all possible* life-lines. Instead, the four-dimensional vector-space can be considered as the larger setting within which this model operates.

## A tetrahedron relates the six demographic time measures

**Fig. 5:**
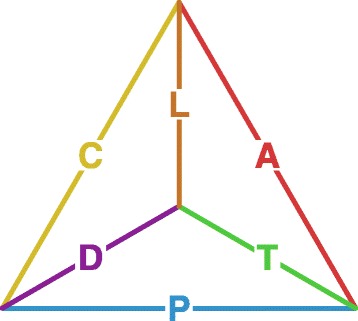
Tetrahedral graph of demographic time hexad identity, with edges labelled by the six time indices

The demographic time framework we present includes three events (period, birth cohort, and death cohort), and it therefore leads to a graph based on the second row and third column of Table 3, here redrawn in Fig. 5 with a slight rearrangement of the vertices, and edges labelled with the six demographic time measures.

There are a total of four triangles in Fig. 5, one for each of the triad sub-identities, such that each time measure is an element of two triad identities. Each of these triangles is the edge-graph of a face of the tetrahedron, ergo each face of the tetrahedron represents one of the triad identities. It is reasonably straightforward to imagine this graph as the wire-frame of a 3-d tetrahedron—as the 3-d edge structure of the tetrahedron platonic solid.

For exposition, imagine that the middle vertex of Fig. 5 is the top (closer to the eye), while the outer edges A, P, and C form the base of the tetrahedron, forming the much-studied APC identity. The South face corresponds to the TPD identity, the Northeast face to the TAL identity, and the Northwest face to LCD identity. The transformation of this three-event system to a three dimensional space follows Definition 2. It may also suffice to simply imagine that each face of the tetrahedron forms the basis of a plane, such that the tetrahedron itself defines four planes. These four planes are the four temporal planes that underly the four diagrams presented in “The triad identities” section. A full diagram of the demographic time identity (or any identity based on three events) conforms in this way with the geometry of a tetrahedron.

## Diagram of the hexad identity

There are different ways to proportion this three-dimensional construct, of which we only present the isotropric mapping^3^. In an isotropic projection, the tetrahedron is regular, such that all edges are of the same length, and the units of each of the six represented time measures are therefore equal. In this case, the four triad identities map to their respective temporal planes as tessellations of equilateral triangles. When the plane parallel to each respective face is repeated in equal intervals, we have an isotropic 3-d space^4^. Displaying all planes simultaneously creates a very dense and difficult-to-read diagram. We opt to delineate the space using the intersection of two planes.

**Fig. 6:**
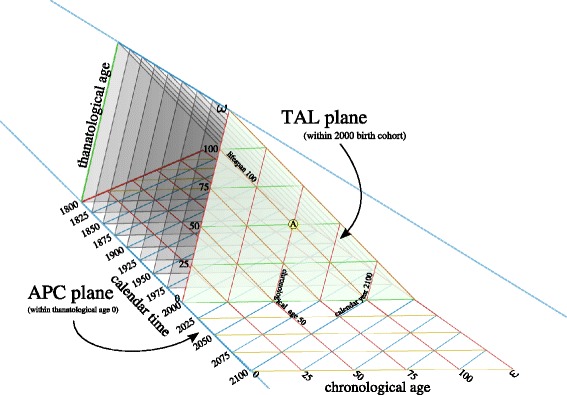
Diagram of the hexad identity, showing a sequence of TAL planes intersecting with a single APC plane at the base

Figure 6 gives a view of a demographic time diagram that corresponds to the hexad identity, where birth-cohort TAL cross-sectional planes are placed in sequence in a perspective drawing^5^. The most recent TAL plane, for the year 2000, is placed in the front, whereas past TAL planes are stacked behind it, highlighted in 25-year intervals. The left edge of the frontmost TAL plane is labelled as an axis for thanatological age, although the same tick marks also serve for completed lifespan. The base of this figure is the APC plane, drawn through thanatological age 0. Each of the TAL planes sits atop a single birth cohort line from the familiar APC plane that makes up the base of the diagram.

For example, imagine an infant born in the year 2000. Without further information, we only know that this infant is located somewhere on the thanatological age axis (left edge) of the front TAL plane. If this infant is destined to die in the year 2100, then the vertical position at birth will be at the axis tick for thanatological age 100. This person’s entire life stays on the 100 lifespan line (labelled), descending over time towards thanatological age 0 at the base. Point A marks the midpoint in life for this individual, at chronological age 50 (red line, labelled), and thanatological age 50 (green line). If another APC plane were drawn through thanatological age 50, we would see that point A is in the year 2050. Since all individuals born in the year 2000 complete the same age in the same year, we can also recuperate the year for point A by following the chronological age 50 line (red) down to where it meets the blue line for the year 2050. The lifeline descends downward towards the APC plane for thanatological age 0 at chronological age 100, meeting the year 2100, which is individual A’s death cohort.

The density and location of imaginary lifelines in this diagram, omitting migration, is purely a function of birth cohort size and survival. For extinct cohorts all lifelines can be positioned, but for the 2000 birth cohort this is not yet the case. Most of the front TAL plane is in the future. One may imagine yet another plane intersecting this space—the “present plane,” which is identical to the period TAL plane for the present moment. To see how this plane divides the space, imagine that we are in the year 2025, and follow the blue line in the APC base inward 25 years to where it meets the red line for chronological age 25, and follow the red line up the front TAL plane. A single plane cuts through the year 2025 and chronological age 25 from the year 2000 birth cohort. This plane shifts forward or backward in time to meet the present year. In this particular plane, the coordinates T, L, and D are uncertain. The period TAL plane *ω* years in the past is fully identified, ergo, theoretically the lifespan of each individual in the time of Lexis is knowable.

Figure 6 could have been drawn with TPD or LCD planes highlighted as well, but these can still be imagined upon the current rendering. TPD planes transect this space through any given chronological age, for instance. Imagine a wall on the left side of the prism, cutting through chronological age 0 (recall Fig. 2). In this case, the thanatological age axis is indicated in the very back of the diagram, calendar time becomes another axis, and death cohort diagonals are not drawn. TPD planes sequence inward from this first plane, always forming cross-sections through chronological age. The LCD plane is to be found by rotating the current prism such that the angle of view is directly orthogonal to lifelines, which would then appear as points (recall Fig. 4).

The essential property of this perspective diagram is that lifelines start and end in parallel, desceding downward and forward in time. A real population of renewing lives, spread over time and over the typical range of human lifespans, will tend to fill the entirety of the prism depicted in Fig. 6, and any given point in the prism can be given six demographic time coordinates, of which three are redundant. A similar 3d construct could be made for any hexad time-identity, and these are not strictly limited to event-duration identities based on three events.

## Application

The coordinate system described here may be useful for the visualization of data, to enable discovery, and to better inform demographic methods. We have not yet mentioned how such developments might arise in practice. We therefore give a brief case study to demonstrate the potential of the present framework, but this is far from an exhaustive application of its usefulness for other substantive questions, nor is the case study described in complete rigor. Specifically, we reason that projections or comparisons of prevalence-based healthy life expectancy (HLE) are in many cases biased in period prevalence-based models unless one takes into account the thanatological age pattern of prevalence, as well as mortality differences.

There are three steps in our empirical inquiry. The first step is to visualize variables on health outcomes using the demographic time diagram. The second step is pattern detection. We assess the primary time measures over which health outcomes appear to vary. Under the assumption that these patterns of temporal variation are empirically regular, we describe a method of standardizing health expectancy calculations for morbidity conditions whose prevalence is more closely related to thanatological age. Finally, we reason that period estimates of health expectancies for certain health conditions are biased when mortality has been or will-be changing, and comparisons of HLE between populations with different mortality are also biased. We conclude that comparisons of health expectancies might be biased in ways not previously documented.

Let us take the example of self-reported health (SRH). The data come from the RAND version of the US Health and Retirement Study (Health and Retirement Study 2013; RAND 2013). Since this survey includes multiple dated observations of individuals, as well as information on time of birth and a followup for time of death, we have or can derive each of the six demographic time measures for each observation. Further methodological details are given by (Riffe et al.: Time-to-death patterns in markers of age and dependency, forthcoming). We opt to view data on TAL surfaces because these allow us to judge the shape of prevalence over the life course, specifically to show how SRH prevalence variation is summarized more efficiently as a time-to-death pattern than as an age pattern.

**Fig. 7:**
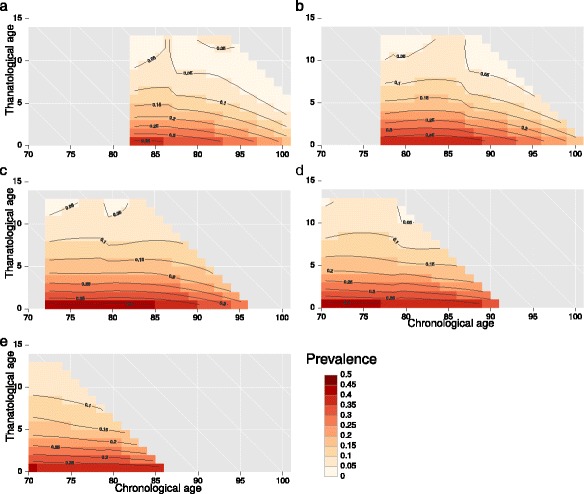
Prevalence of males self-reporting poor health by chronological and thanatological age, by quinquennial birth cohorts, 1905–1925 (Sources: Health and Retirement Study 2013; RAND 2013)

Figure 7 displays a series of TAL surface plots of male SRH prevalance, each referring to a different quinquennial birth cohort (1905–09, 1910–14, …). These follow the coordinates of the TAL diagram in Fig. 3. The *x*-axis is chronological age, the *y*-axis is thanatological age, and downward-sloping diagonals delineate lifespan. Lifelines (not drawn) descend parallel to the downward diagonals seen on the background grid. The density of lifelines in each surface is not visible in this rendering, but one can imagine the mode of the lifetable deaths distribution running down the diagonal that meets near age 80 on the *x*-axis. Each surface describes the end-of-life SRH prevalence of a birth cohort for the range of lifespan permitted by the survey, but with a lower bound of 70 and an upper bound of 100. Surfaces are therfore shifted down five ages (leftward) for each successive quinquennial birth cohort. Colors and contours indicate prevalence value ranges, with pastel pink for low values (under 10%) and deep reds for high values (over 40%).

Contour lines in the surfaces are perpendicular to the primary direction of variation. For each cohort, the deepest red bar is located in the last year of life and spread over a wide range of ages, giving a roughly horizontal contour line. Other contour lines are also relatively horizontal. This means that variation (in this window of observation) is mainly over thanatological age and not over chronological age. Were variation mostly a function of chronological age the contour lines would be vertical. For each of these birth cohorts we have a series of prevalence trajectories—empirical examples of the lifeline morbidity trajectories often conceptually diagrammed in the literature on morbidity compression (e.g., (Fries 2005)). If we were to summarize each of these surfaces with a single line, a thanatological age pattern would give a much more compact description than a chronological age pattern. Patterns are also relatively stable between cohorts.

When weighted by lifelines, the marginal chronological age pattern of SRH, i.e., as measured with the “Sullivan curve” (Sullivan 1971), would show an increasing tendency over age, in agreement with common expectations. However, such an increasing pattern over age is a marginal artifact, due to an interaction between the distribution of lifespans and the relatively fixed underlying pattern of morbidity seen in Fig. 7. These surfaces can indeed be tidily summarized with a single line, but it is a line over the thanatological age margin rather than over chronological age.

Since the patterns for each of these cohorts can be presumed to be the same, any shifting in the distribution of age at death ought not produce a change in the expected years of poor health for a given length of life. Further, cohort expected life years spent in poor health should also be approximately the same, even if the underlying age-at-death distribution shifts upward. If morbidity change is a pure function of thanatological age, an increase in life expectancy should increase healthy life expectancy by the same amount. This is not the prediction when we base analyses on the chronological age pattern of self-reported health. Indeed, an underlying morbidity pattern as stable as that seen in Fig. 7 would predict improvements in the marginal chronological age pattern of self-reported health if the lifespan distribution were to shift to higher ages. This is because a higher age at death implies more years lived in ages farther from death, where prevalence is low. This potential bias in the current status quo of morbidity measurement and prediction leads to pessimistic morbidity scenarios when mortality improvements are projected, and it undermines health expectancy comparisons between groups with different mortality (Riffe et al. 2016). Cohort health expectancies are in either case unbiased, but these are also not commonly estimated due to data constraints. This approach and essential finding is in agreement with the results of similar analytic approaches to the prediction of healthcare expenditure(e.g., Geue et al. 2001, Miller 2014).

Using the data from our example surfaces, we calculate some basic results that support our case. Let us take the population of US males aged 60 and older, and assume that mean time-to-death trajectory derived from the Fig. 7 surfaces is valid for them. We apply this trajectory to the synthetic stationary population of each year from 1980 and 2010 (Human Mortality Database 2014) following the formulas in (Riffe et al. 2016). We then calculate the resulting healthy and unhealthy life expectancies, and compare these with expectancies calculated using the standard Sullivan method and assuming the 1980 chronological age pattern of poor SRH^6^. Total remaining life expectancy at age 60 increased 4.3 years from 17.4 in 1980 to 21.7 years in 2010. Assuming the time-to-death prevalence trajectory, we calculate healthy life expectancies of 15.7 and 19.9, respectively, an increase of 4.2 years. Unhealthy life expectancy in this scenario increased just 0.1 years. Had we used the Sullivan curve from 1980 to calculate the 2010 values, we would have predicted an increase of 0.7 years in unhealthy life expectancy, or 39% versus the 4% “observed” in this simple scenario.

This is a large difference in projected morbidity, and it is based on a relatively minor tweak to standard methodology, itself inspired by viewing data under the conditions enabled by the demographic time framework and adjusting standard demographic methods to capture the direction of temporal variation in data. There is a wide variety of prevalence patterns when viewed in this way (Riffe et al.: Time-to-death patterns in markers of age and dependency, forthcoming), (Wolf et al. 2015), and much empirical and methodological work is still required to verify that these findings are representative and to understand the consequences for the standard ways of comparing and projecting HLE. Our objective in this application has been to demonstrate how viewing data structured by the time-framework we propose can lead to new understandings and approaches to processes over the life course. Other methodological applications of this framework are imaginable in other phases of the life course, or non-human subjects.

## Conclusions

The age-period-cohort relationship is a special subset of a richer and unbounded set of potential time identities. Of this infinite set of temporal relationships, we present one six-way demographic time identity that expands the Lexis diagram to a Lexis “space” so as to structure transitions with respect to both birth and death (entry and exit). We call this hexad relationship a demographic time framework because it is based on the events of birth and death in calendar time, entailing six time measures: chronological age (A), period (P), birth cohort (C), time to death (T), death cohort (D), and individual lifespan (L). In the “From dyads to the triad identities” section, we show how combinations of these time measures imply four triad identities, each of which consists in simple linear relationship between its three constituent time measures. We describe how each triad identity can be extended into a temporal plane, with a characteristic diagram. The four triad identities underly a family of four diagrams that include the familiar Lexis diagram, but also three either new or uncommon diagrams: The TPD, which is a sort of dual to the Lexis diagram; the TAL, whose use we deomonstrate in the “Application” section; and the LCD diagrams.

These four identities and diagrams relate to one another in a single relationship that can be represented in three-dimensional space. In the “Diagram of the hexad identity” section we render a diagram of the demographic time identity. We argue that the full three dimensional diagram is not necesarily a practical way to represent demographic data, but that it forms a useful reference to understand demographic structure. Practically, data structured by all six demographic time measures can be represented on any of the four diagrams if controlled properly. In the “Application” section we present a brief application of this technique to the prevalence of poor self-reported health in older ages in the USA. We show that the choice of age pattern when calculating prevalence-based measures of healthy life expectancy can have a large impact on healthy life expectancy. The size of the effect varies depending on the morbidity pattern, and on how fast mortality and morbidity are both changing. However, the experience of old-age mortality improvement in recent decades leads us to suspect that many projections of old-age morbidity burden are likely to be needlessly pessimistic if it is the case that the prevalence of pertinent health conditions varies primarily as a function of time to death. This clearly merits further study in the case of human population health, and we speculate that findings of similar import may arise if this framework is used to visualize data and inform new methods in other unrelated areas of investigation.

In “The relationship between events and durations” section, we digress to present a more general event-duration identity framework, which allows us to situate the demographic time hexad identity more rigorously as a special case of an event-duration framework. We compare this identity with other relatively complicated temporal relationships in the literature, including the Lexis (1875) marriage identity and the illness-death model by Brinks et al. (2014). Our comparison between complex statistical designs serves to illustrate the transferability of the concepts we present to other applications. The examples we select to illustrate this framework happen to be from social and medical sciences, but the same relationships hold in any single or multi-state situation. That is to say, one may represent the time-space of any phenomenon, transition, dated event, or sequence thereof by deriving the time identity graph as in Table 3 and using this as the basis of further analysis.

Data visualization is an effective way to detect patterns in temporal variation. The generalized time framework we propose is conceived as one adequate to capture all possible temporal variation. The demographic time hexad identity is a special case whose use we suggest for visualizing macro patterns in demographic data, probably via small multiples of successive time slices in one of the diagrams from the “From dyads to the triad identities” section, similar to that shown in Fig. 7 on the basis of the TAL diagram. Such visualization strategies at this time are exploratory, and this is a technique that may benefit from further refinement. Further, a cross-section through the demographic time-space need not be parallel to one of the four identity-planes. Other more complicated temporal designs are also possible, potentially based on even higher dimensional time-spaces. In this case, cross-sections may also be a helpful trick for visualization, although this is an area in need of future work. Further, if the purpose of visualization in this case is merely to detect the principal direction of variation, appropriate statistical methods should be developed (or recommended) to do so in a more rigorous way, and these should be flexible with respect to the full set of time measures implied by a given identity.

Several lines of substantive research may be augmented by or based on the findings we present. For example, it is of public health interest to document the full range of late-life morbidity patterns over various time measures. We do not at this time know how the late life morbidity patterns referred to in the “Application” section change over time or vary between populations, for example. This has implications for the use of health expectancies and related measures in comparative studies of disease burden. However, the time framework we describe may also be useful more generally to structure disease processes. More broadly, the temporal dynamics of classiscal demographic processes such as childbearing, partner formation and dissolution, migrations, employment transitions, and temporal interactions between these events may also be fully captured and explored under our framework. To do so, we offer some tentative advice: First, create the identity graph of interest; next, complete the data to include “implied” time measures; then, toggle through cross-sections of the structured data to determine which ones reveal important patterns. The phenomena that we are most likely to learn the most about by taking this simple analytic step are those that are dogmatically held to vary only over age.

Finally, we believe in the pedagogical value of the framework introduced in this paper. We hope that the present inquiry will be useful as a teaching instrument in the same way as Lexis diagrams have formed a part of basic demographic education. Our generalized time framework and the relationship between the six dimensions of demographic time both help situate the APC paradigm in a broader context. Just as scientific discovery in general depends partly on the development of finer optics and instrumentation, we hope that the framework we describe will prove an instrument to enable new discoveries in formal and empirical demography, as well as other diverse fields of investigation.

## Endnotes

^1^ See e.g., Keiding (2011) for an overview of that literature.

^2^ For points in time past the time at diagnosis *d*_2,3_ becomes negative and can be interpreted as time since diagnosis.

^3^ To compare, Lexis (1875) used a Cartesian mapping for his marriage identity, with right angles between birth cohort, age at marriage, and duration married.

^4^ The isotropic space that results from this framework is known in other disciplines with different nomenclatures. In geometry, this structure is called the tetrahedral-octahedral honeycomb, a variety of space-filling tessellation. In architecture, it is found in the octet truss system. In physics it is called the isotropic vector matrix. Constructs following this geometry exist in nature, in other theoretical settings, and in man-made structures.

^5^ The coordinates used to render Fig. 6 are isotropic. However, there are no 60° angles in this figure due to the use of parallax and an indirect viewing angle in this rendering for the sake of increased legibility.

^6^ The 1980 chronological age pattern of poor SRH is calculated from the 1980 stationary population and the same fixed time-to-death prevalence trajectory.
